# Melatonin protects against defects induced by Enniatin B1 during porcine early embryo development

**DOI:** 10.18632/aging.202484

**Published:** 2021-02-11

**Authors:** Xiangyu Wang, Mingju Sun, Jingyu Li, Xuexiong Song, Hongbin He, Yanjun Huan

**Affiliations:** 1 College of Veterinary Medicine, Qingdao Agricultural University, Qingdao 266109, Shandong Province, China; 2 Chongqing Key Laboratory of Human Embryo Engineering, Chongqing Health Center for Women and Children, Chongqing 400013, China; 3 College of Life Science, Shandong Normal University, Jinan 250014, Shandong Province, China

**Keywords:** Enniatin B1, melatonin, DNA methylation, embryo, pig

## Abstract

Exogenous factors influence embryo development. Enniatin B1 (EB1), one emerging mycotoxin of Fusarium fungi, can cause damage to cells and mouse blastocysts. However, the toxicity of EB1 on porcine embryo development and whether melatonin can eliminate the detrimental effects of EB1 on embryos remain unclear. Here, this work demonstrated that EB1 significantly decreased the cleavage and blastocyst rates and blastocyst cell number of embryos in a dose and time dependent manner. Further study displayed that EB1 obviously destroyed nuclear remodeling dynamics. Importantly, EB1 triggered embryo apoptosis through downregulating the expression of *Sod1,* *Gpx4*, *Cat* and *Bcl2l1* while upregulating the transcription of *Bax* and *Caspase3*. Moreover, EB1 significantly disrupted the transcription of *Dnmt1*, *Dnmt3a*, *Tet1* and *Tet3*, further leading to incomplete DNA demethylation of *CenRep*, *Oct4*, *Nanog* and *Sox2*, thus, the expression of *Eif1a*, *Oct4*, *Nanog* and *Sox2* remarkably decreased. Whereas EB1-exposed embryos were treated with melatonin, these disorders were obviously ameliorated, and the development ability of embryos was also rescued. In conclusion, EB1 exerted detrimental effects on porcine early embryos, while melatonin effectively rescued EB1-mediated defects in embryos. This work provides a novel insight into the improvement of embryo quality and the promotion of human and animal reproduction.

## INTRODUCTION

The application of embryo engineering technology, such as artificial insemination, *in vitro* fertilization, and embryo transfer etc., has accelerated the development of human and animal reproduction [1, 2]. However, early embryos, both *in vivo* and *in vitro*, are extremely vulnerable to exogenous factors. For example, food or feed contaminated with toxins degrades early embryos, while antioxidants promote the development of embryos [3, 4]. Thus, exogenous factors have attracted the extensive attention and been designed to enhance embryo development.

Enniatin, one kind of emerging mycotoxins, is a secondary metabolism product produced by Fusarium fungi, and has been closely focused in recent years [5, 6]. To date, a variety of natural enniatin analogues has been identified, and Enniatin A, A1, B and B1 are the most common in food or feed products [5, 7]. Previous studies have shown that Enniatins exert cytotoxin, inhibiting cell proliferation, and induce oxidative stress, causing cell apoptosis or death [5, 8, 9]. Enniatin B1 (EB1) is also proven to trigger cell apoptosis and exert embryotoxicity on mouse blastocysts [9, 10]. However, the potential toxicity and mechanism of EB1 during early embryo development remain unclear.

Accumulated studies have demonstrated that the negative effects of exogenous hazardous compounds on embryos are embryotoxicity, apoptosis or epigenetic modification damage [10–12]. During embryogenesis, nuclear remodeling, referring to nuclear decondensation, formation and fusion of paternal and maternal pronucleus, and nuclear division, occurs in the first cell cycle, and is necessary for the normal development of early embryos [13]. Then, the delayed or incomplete nuclear remodeling induced by hazardous compounds would cause embryo development arrested. As for embryo apoptosis, it is a key marker to evaluate embryo quality, and inappropriate trigger by deleterious factors has been proven to induce embryotoxicity and impair embryo quality [4, 10]. Moreover, epigenetic modification, especially DNA methylation, which predominantly occurs at the fifth carbon of cytosine, is also affected by exogenous compounds [14]. During early embryo development, DNA methylation reprogramming, mainly regulated by DNA methyltransferases (Dnmts) and ten-eleven translocation (Tet) dioxygenases, is essential for the normal expression of genes related to embryo development, however, this progress is vulnerable to damage by exogenous hazardous compounds, then, the destroyed DNA methylation reprogramming leads to the disturbed expression patterns of genes, especially pluripotent genes, and further the impairment of embryo development competence and quality [14, 15]. EB1, as one emerging mycotoxin, could also take the similar toxic effect on embryo development. Thus, to reveal these potential detrimental characteristics of EB1 would help to provide the appropriate strategy to protect embryo development and quality.

To overcome the detrimental effects of hazardous compounds on embryos, antioxidants are usually applied, and melatonin, a well-known free radical scavenger, is proven to stimulate antioxidant-related gene expression, reduce apoptosis, and promote embryo development [16–18]. Recently, studies have demonstrated that melatonin participates in DNA methylation reprogramming during embryo development [19–21]. Moreover, melatonin is also shown to protect embryos against various exogenous hazardous factors [22–24]. Then, melatonin is speculated to ameliorate the detrimental effects of EB1 on embryos, however, to our knowledge, no related studies have investigated the potential protective role of melatonin against EB1 mediated defects during early embryo development.

In this study, porcine early embryos were employed to investigate the detrimental effects of EB1 and the antagonistic role of melatonin against EB1 during the development progress. The results displayed that EB1 exerted the detrimental effects on the development competence and quality of embryos through destroying nuclear remodeling, apoptosis, DNA methylation reprogramming and gene expression, and also showed that melatonin effectively rescued EB1-mediated defects in embryos. This work provides an important support for human and animal reproductive health.

## RESULTS

### Effect of EB1 on the development of early embryos

**Figure 1:**
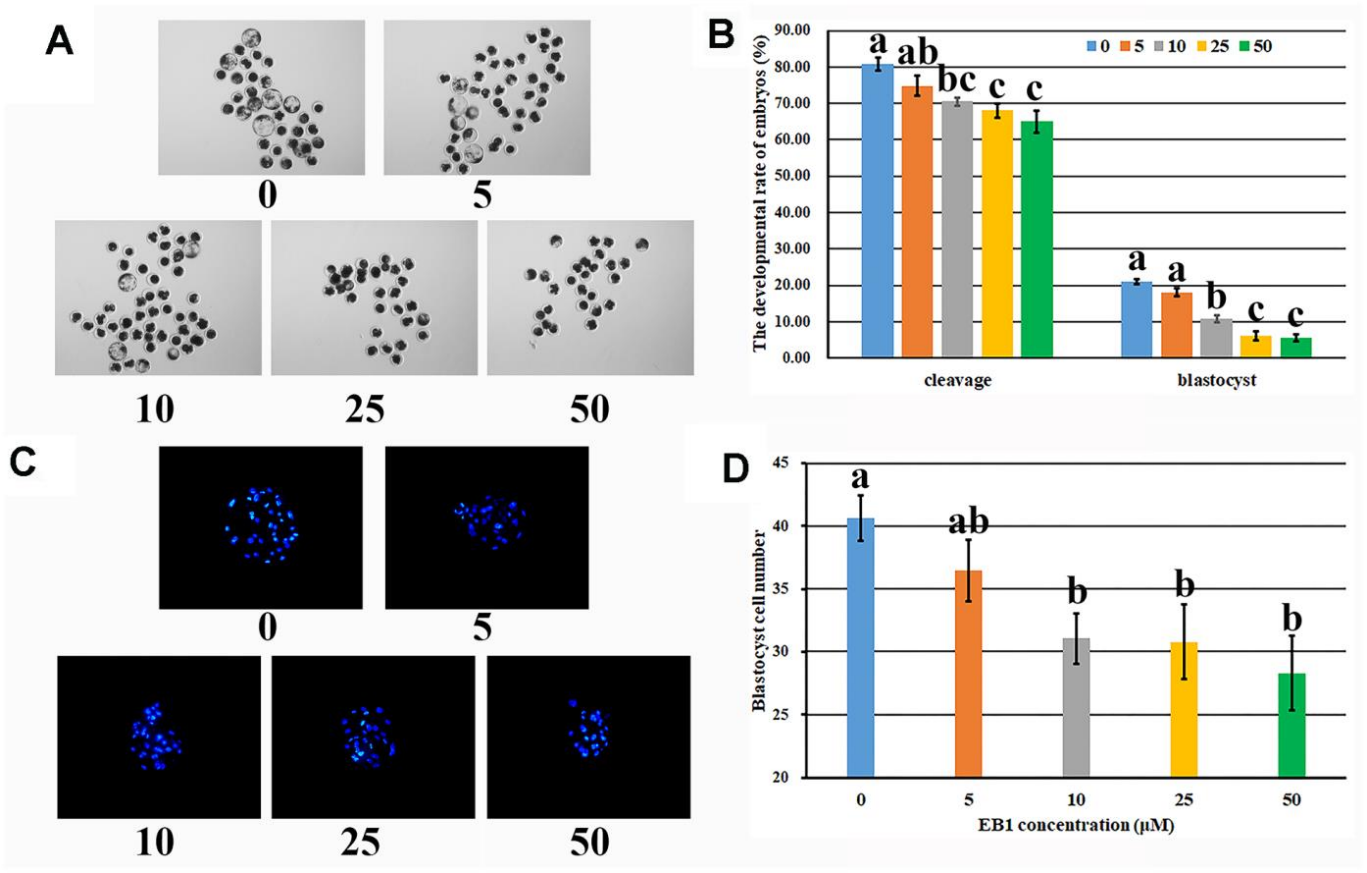
**Effect of EB1 with different concentrations on early embryo development.** (**A**) blastocyst (× 40). (**B**) cleavage rate and blastocyst rate. (**C**) blastocyst cell number (× 200). (**D**) average blastocyst cell number. ^a-c^Values for a given group in columns with different superscripts differ significantly (p < 0.05).

To clarify the detrimental effects of EB1 on early embryo development, the dose and time dependent manners of EB1 were investigated. The results displayed that the development competence of early embryos was reduced after EB1 treatment with different concentrations, and 10, 25 or 50 μM EB1 resulted in the significantly lower cleavage and blastocyst rates and blastocyst cell number compared with the untreated group (Figure 1 and Supplementary Table 3, P<0.05). Considering that 25 or 50 μM EB1 resulted in the even more significantly reduced blastocyst rate and was the high dose for early embryos, 10 μM EB1 was chosen for the subsequent treatment. For analysis of the time effects of EB1 on early embryos, when the treatment time was shortened to 12 h, no significant development differences were observed compared with the untreated group, whereas the rates of cleavage and blastocyst were significantly higher than those of embryos treated for 24 h (Supplementary Table 4, P<0.05). Thus, EB1 exerted the detrimental effects on early embryos, and treating embryos with 10 μM EB1 for 24 h significantly reduced the development competence and quality of embryos.

### Effect of melatonin on the development of EB1-exposed embryos

**Figure 2:**
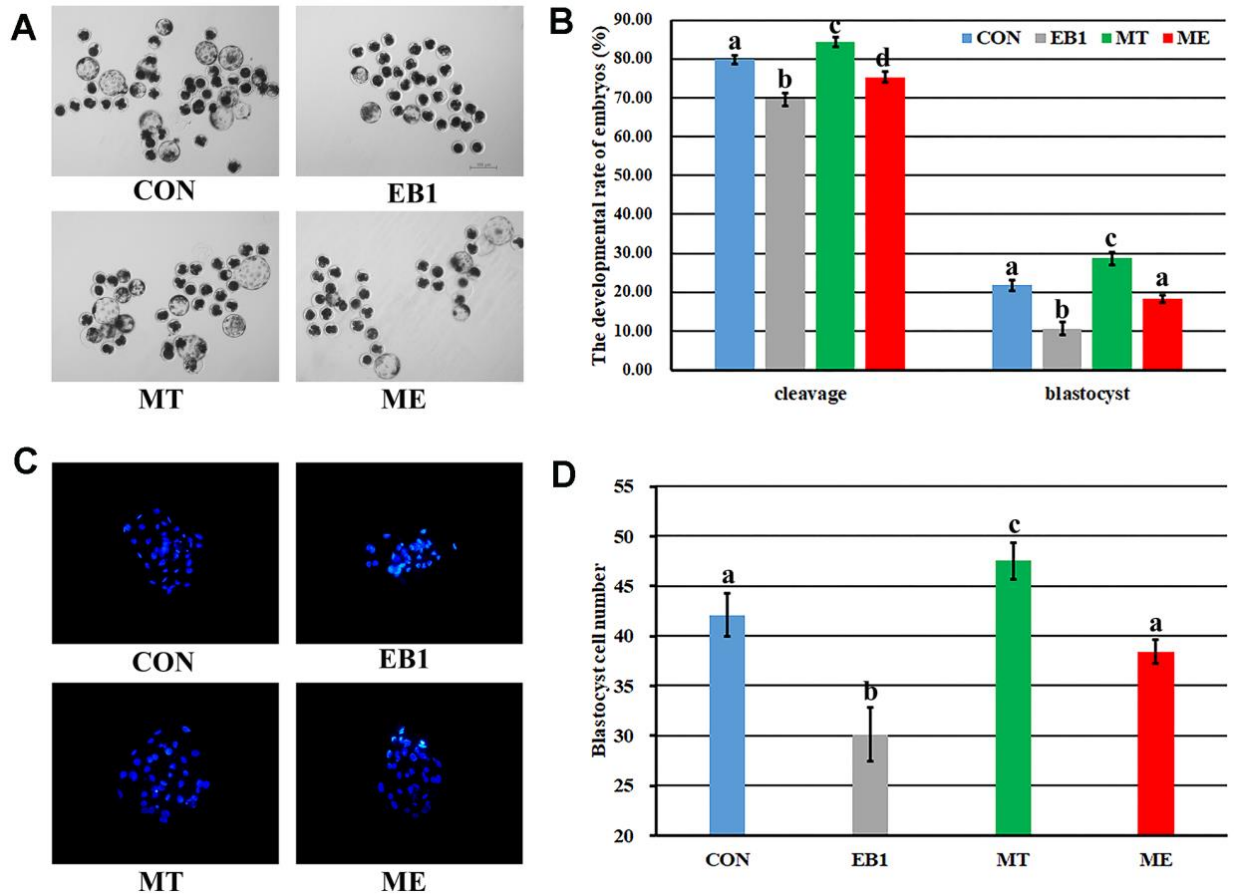
**Effect of melatonin on the development of EB1-exposed embryos.** (**A**) blastocyst (× 40). (**B**) cleavage rate and blastocyst rate. (**C**) blastocyst cell number (× 200). (**D**) average blastocyst cell number. CON, the control group. EB1, embryos treated with EB1. MT, embryos treated with melatonin. ME, embryos treated with both melatonin and EB1. ^a-d^Values for a given group in columns with different superscripts differ significantly (p < 0.05).

To overcome the detrimental effects of EB1 on early embryos, melatonin was also added to treat embryos. As shown in Figure 2 and Supplementary Table 5, EB1 significantly reduced the cleavage and blastocyst rates and blastocyst cell number, while melatonin (MT group) significantly upregulated the cleavage and blastocyst rates and blastocyst cell number compared with the untreated (CON) group (P<0.05). Moreover, when EB1-exposed embryos were treated with melatonin (ME group), the cleavage and blastocyst rates and blastocyst cell number were significantly higher than those in the EB1 group, and no significant differences of blastocyst rate and blastocyst cell number were observed compared with the CON group though the cleavage rate was still significantly lower (P<0.05). Thus, melatonin effectively protected the development competence and quality of embryos from EB1 exposure.

### Effect of melatonin on nuclear remodeling in EB1-exposed embryos

**Figure 3:**
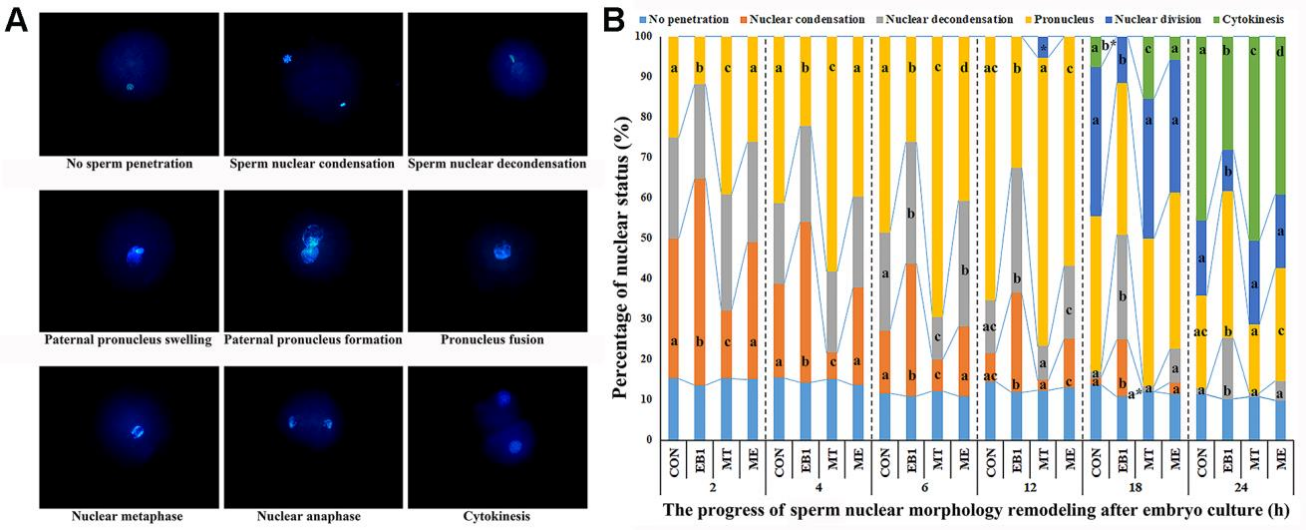
**Effect of melatonin on nuclear remodeling in EB1-exposed embryos during the first cell cycle.** (**A**) embryo nuclear morphology (× 400). (**B**) the percentages of embryo nuclear status. CON, the control group. EB1, embryos treated with EB1. MT, embryos treated with melatonin. ME, embryos treated with both melatonin and EB1. ^a-d^Percentages for a given group in columns with different superscripts differ significantly (p < 0.05).

After sperm penetrated into oocyte, sperm nucleus performed decondensation and swelling and formed paternal pronucleus, which then fused with maternal pronucleus to form the diploid zygote pronucleus, and then zygote carried out mitosis, meaning that nuclear division and cytokinesis successively occurred (Figure 3A). Based on the morphology changes of sperm nucleus, nuclear remodeling progress was mainly divided into sperm nuclear condensation, sperm nuclear decondensation including swelling, paternal pronucleus including formation and fusion with maternal pronucleus, and nuclear division, and oocyte with no sperm penetration and embryo cytokinesis were also examined during the first cell cycle of early embryos (Figure 3B and Supplementary Table 6). In the EB1 group, the significantly upregulated rates of sperm nuclear condensation at 2 h, 4 h, 6 h, 12 h and 18 h, sperm nuclear decondensation at 6 h, 12 h, 18 h and 24 h, and paternal pronucleus at 24 h, and downregulated rates of paternal pronucleus at 2 h, 4 h, 6 h and 12 h, nuclear division at 18 h and 24 h, and cytokinesis at 18 h and 24 h were observed, while the MT group displayed the significantly lower percentages of sperm nuclear condensation at 2 h, 4 h and 6 h and sperm nuclear decondensation at 6 h, and higher percentages of paternal pronucleus at 2 h, 4 h and 6 h, nuclear division at 12 h, and cytokinesis at 18 h and 24 h compared with the CON group (P<0.05). Importantly, the ME group showed the significantly downregulated rates of sperm nuclear condensation at 2 h, 4 h, 6 h, 12 h and 18 h, sperm nuclear decondensation at 12 h, 18 h and 24 h, and paternal pronucleus at 24 h, and upregulated rates of paternal pronucleus at 2 h, 4 h, 6 h and 12 h, nuclear division at 18 h and 24 h, and cytokinesis at 18 h and 24 h compared with the EB1 group, and only the significantly increased percentage of sperm nuclear decondensation at 6 h, and decreased percentages of paternal pronucleus at 6 h and cytokinesis at 24 h compared with the CON group (P<0.05). All these results demonstrated that EB1 delayed nuclear remodeling progress, while melatonin ameliorated the disrupted nuclear remodeling induced by EB1 during the first cell cycle of early embryos.

### Effect of melatonin on apoptosis in EB1-exposed embryos

**Figure 4:**
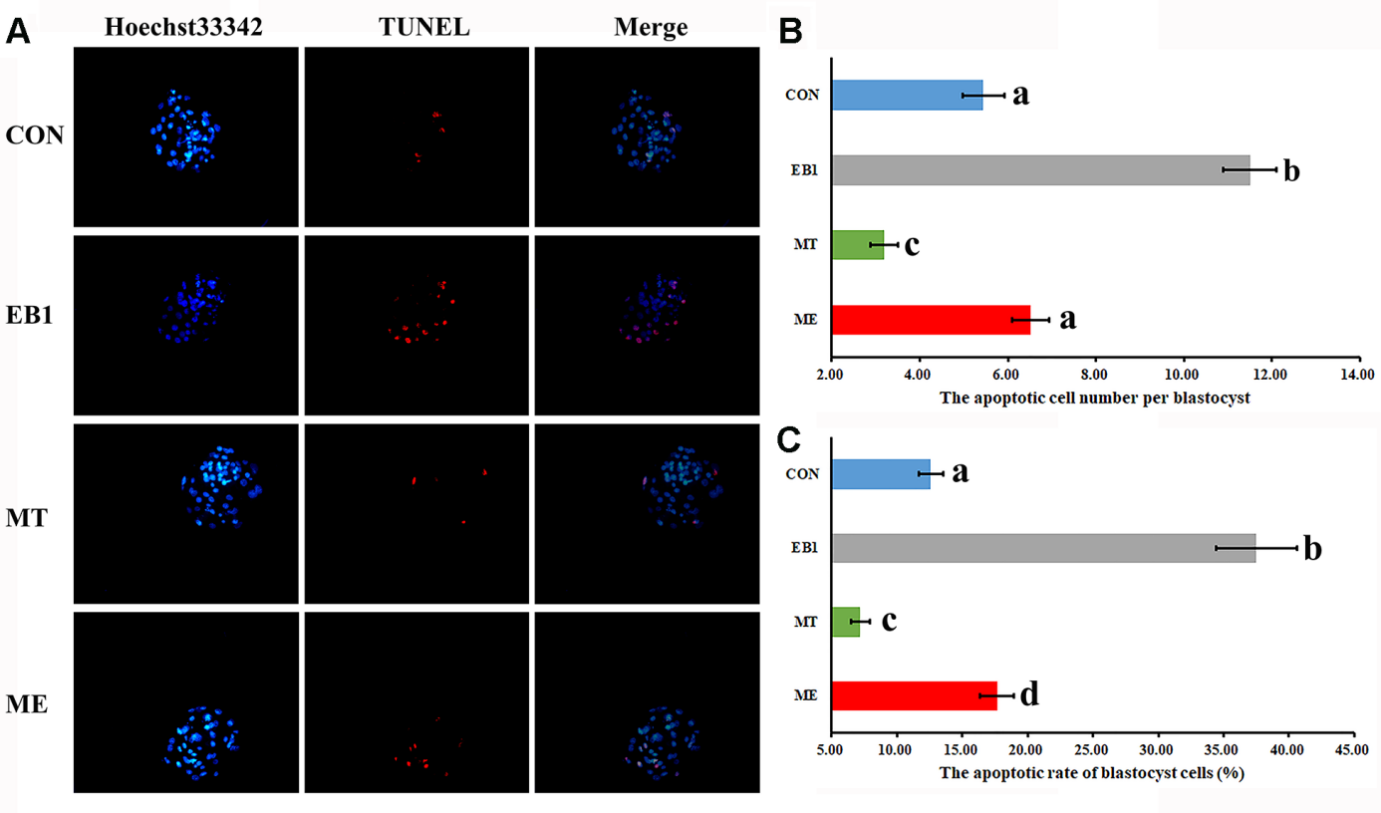
**Effect of melatonin on apoptosis in EB1-exposed embryos.** (**A**) blastocyst cell apoptosis status (× 200). (**B**) average apoptotic cell number per blastocyst. (**C**) blastocyst cell apoptotic rate. The number of blastocysts for apoptosis detection in the CON, EB1, MT or ME group was 29, 16, 32 or 25, respectively. CON, the control group. EB1, embryos treated with EB1. MT, embryos treated with melatonin. ME, embryos treated with both melatonin and EB1. ^a-d^Values for a certain group in columns with different superscripts differ significantly (p < 0.05).

**Figure 5:**
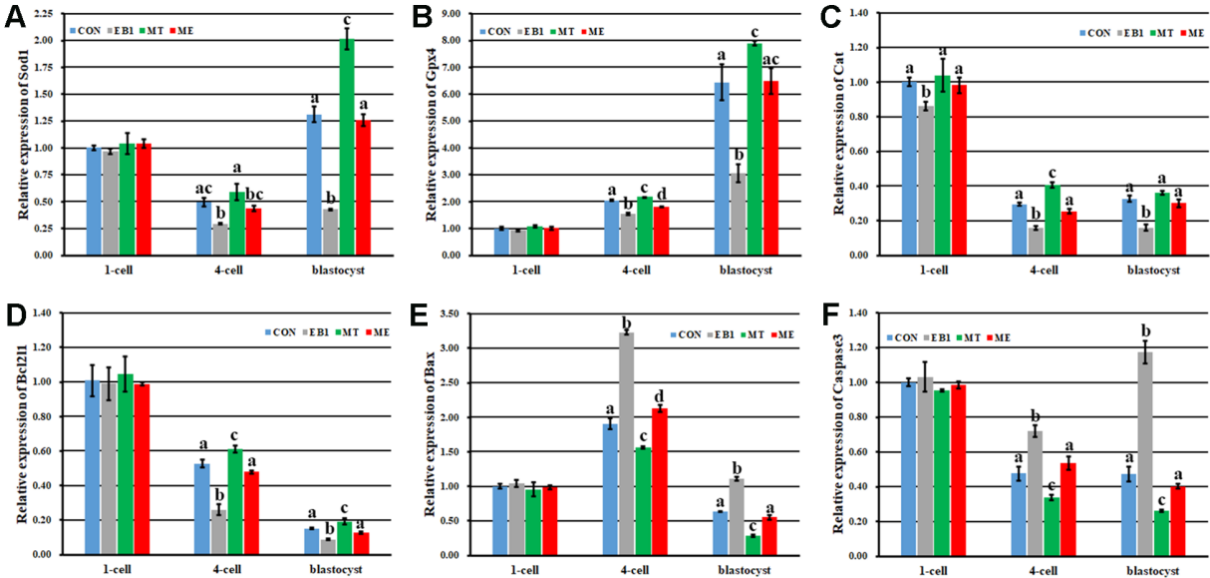
**Effect of melatonin on the expression of antioxidant and apoptosis related genes during the development of EB1-exposed embryos.** (**A**–**F**) relative expression levels of *Sod1*, *Gpx4*, *Cat*, *Bcl2l1*, *Bax* and *Caspase3*, respectively. CON, the control group. EB1, embryos treated with EB1. MT, embryos treated with melatonin. ME, embryos treated with both melatonin and EB1. ^a-d^Values for a given group in columns with different superscripts differ significantly (p < 0.05).

To further explore the antagonistic effects of melatonin on EB1 in early embryos, apoptosis related indexes were analyzed. As shown in Figures 4, 5, compared with the CON group, the EB1 group showed the significantly increased number and rate of apoptotic blastocyst cells, downregulated the expression of antioxidant genes (*Sod1* and *Gpx4*) at the 4-cell and blastocyst stages, antiapoptotic factor *Bcl2l1* at the 4-cell and blastocyst stages and antioxidant gene *Cat* during embryo development progress, and upregulated the transcription of proapoptotic genes (*Bax* and *Caspase3*) at the 4-cell and blastocyst stages, while the MT group displayed the significantly decreased number and rate of apoptotic blastocyst cells, higher expression levels of *Sod1* at the blastocyst stage and *Gpx4*, *Cat* and *Bcl2l1* at the 4-cell and blastocyst stages, and lower the transcription levels of *Bax* and *Caspase3* at the 4-cell and blastocyst stages, respectively (P<0.05). In the ME group, the significantly decreased number and rate of apoptotic blastocyst cells, upregulated expression of *Sod1* at the blastocyst stage, *Gpx4* and *Bcl2l1* at the 4-cell and blastocyst stages and *Cat* during embryo development progress, and downregulated transcription of *Bax* and *Caspase3* at the 4-cell and blastocyst stages were observed compared with the EB1 group, and only the significantly increased rate of apoptotic blastocyst cells, lower expression level of *Gpx4* at the 4-cell stage, and higher transcription level of *Bax* at the 4-cell stage were shown compared with the CON group (P<0.05), suggesting that EB1-induced embryo apoptosis could be effectively blocked by melatonin. Thus, EB1 triggered embryo apoptosis, while melatonin reduced EB1-induced embryo apoptosis.

### Effect of melatonin on DNA methylation reprogramming and gene expression in EB1-exposed embryos

**Figure 6:**
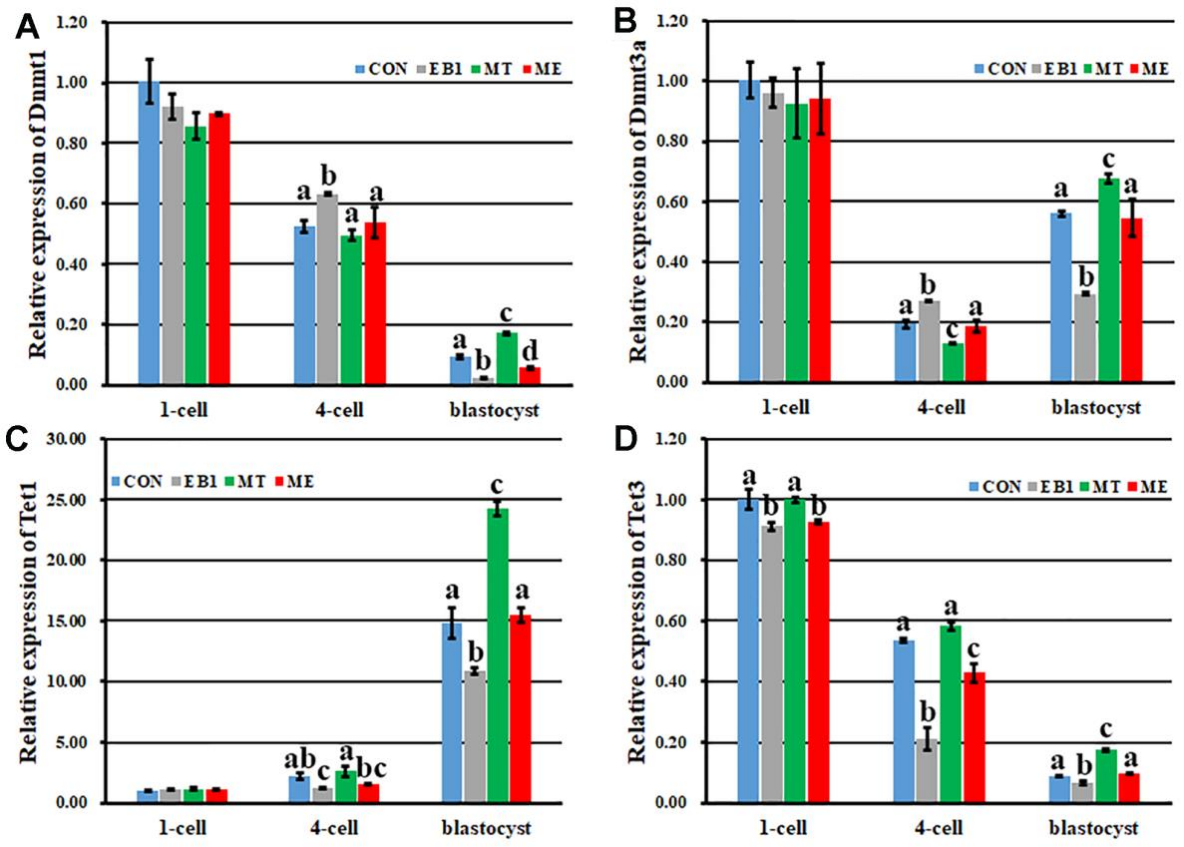
**Effect of melatonin on the expression of DNA methylation reprogramming related genes during the development of EB1-exposed embryos.** (**A**–**D**) relative expression levels of *Dnmt1*, *Dnmt3a*, *Tet1* and *Tet3*, respectively. CON, the control group. EB1, embryos treated with EB1. MT, embryos treated with melatonin. ME, embryos treated with both melatonin and EB1. ^a-d^Values for a given group in columns with different superscripts differ significantly (p < 0.05).

Here, transcription levels of Dnmts and Tets regulating DNA methylation reprogramming were examined in early embryos (Figure 6). The similar expression patterns of *Dnmt1* and *Tet3* with the downward trend, *Dnmt3a* with the first decreased and then increased trend, and *Tet1* with the upward trend during embryo development progress were observed among the CON, EB1, MT and ME groups. When compared with the CON group, the EB1 group showed the significantly higher expression levels of *Dnmt1* and *Dnmt3a* at the 4-cell stage and lower transcription levels of *Dnmt1* and *Dnmt3a* at the blastocyst stage, *Tet1* at the 4-cell and blastocyst stages and *Tet3* during embryo development progress, the MT group displayed the significantly lower expression level of *Dnmt3a* at the 4-cell stage and higher transcription levels of *Dnmt1*, *Dnmt3a*, *Tet1* and *Tet3* at the blastocyst stage, and the ME group only took the significantly lower expression levels of *Dnmt1* at the blastocyst stage and *Tet3* at the 1-cell and 4-cell stages (P<0.05). Importantly, the significantly decreased transcription of *Dnmt1* and *Dnmt3a* at the 4-cell stage and increased expression of *Dnmt1*, *Dnmt3a* and *Tet1* at the blastocyst stage and *Tet3* at the 4-cell and blastocyst stages were observed in the ME group compared with the EB1 group (P<0.05). Thus, melatonin effectively ameliorated the disturbed expression levels of DNA methylation reprogramming related genes in EB1-exposed embryos.

**Figure 7:**
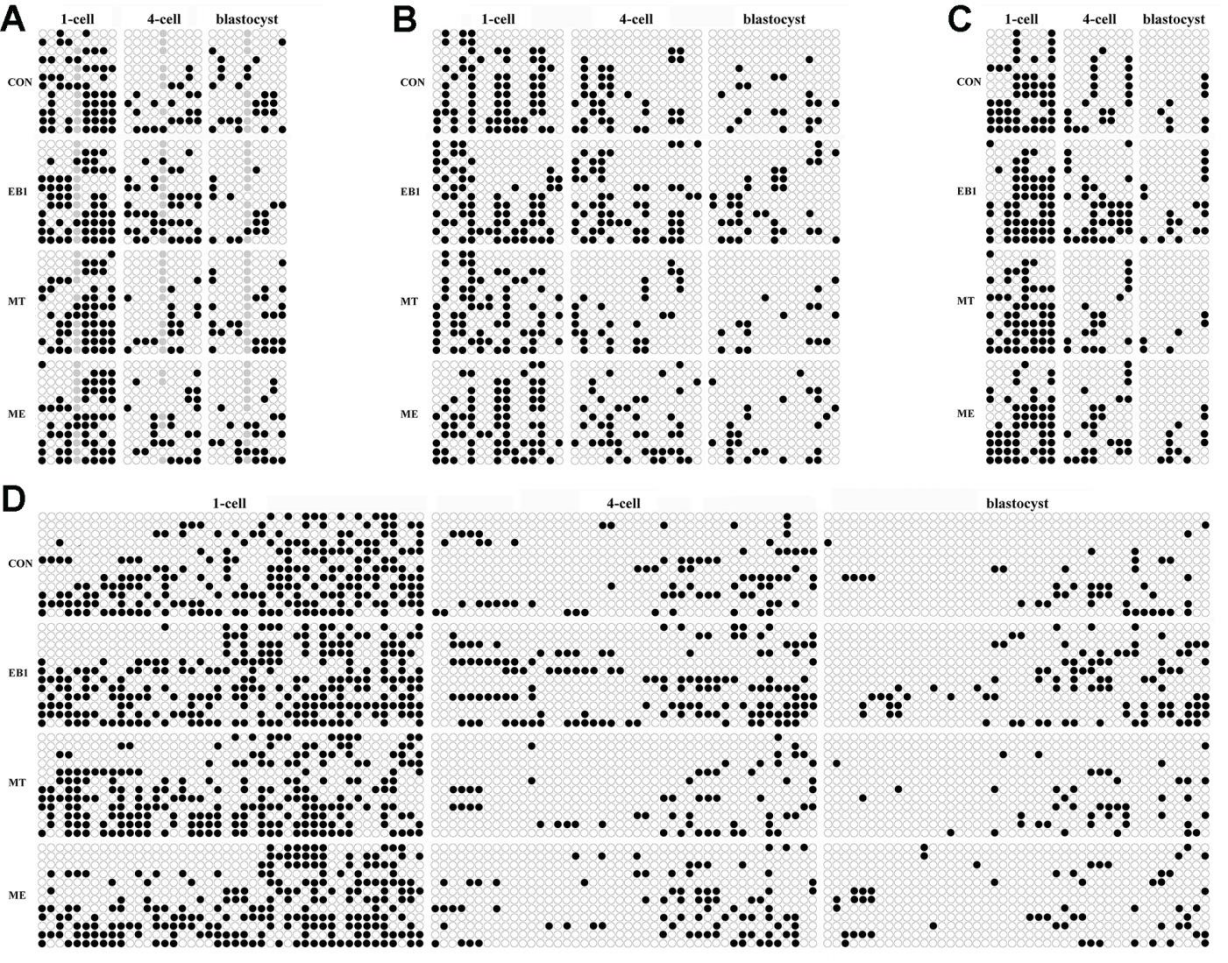
**Effect of melatonin on DNA methylation statuses of genome and pluripotent genes in EB1-exposed embryos.** (**A**–**D**) DNA methylation statuses of genome (*CenRep*), *Oct4*, *Nanog* and *Sox2*, respectively. CON, the control group. EB1, embryos treated with EB1. MT, embryos treated with melatonin. ME, embryos treated with both melatonin and EB1. The black and white circles are the methylated and unmethylated CpG sites, respectively, and the gray circles represent the mutated and/or single nucleotide polymorphism (SNP) variation at the certain CpG sites.

**Figure 8:**
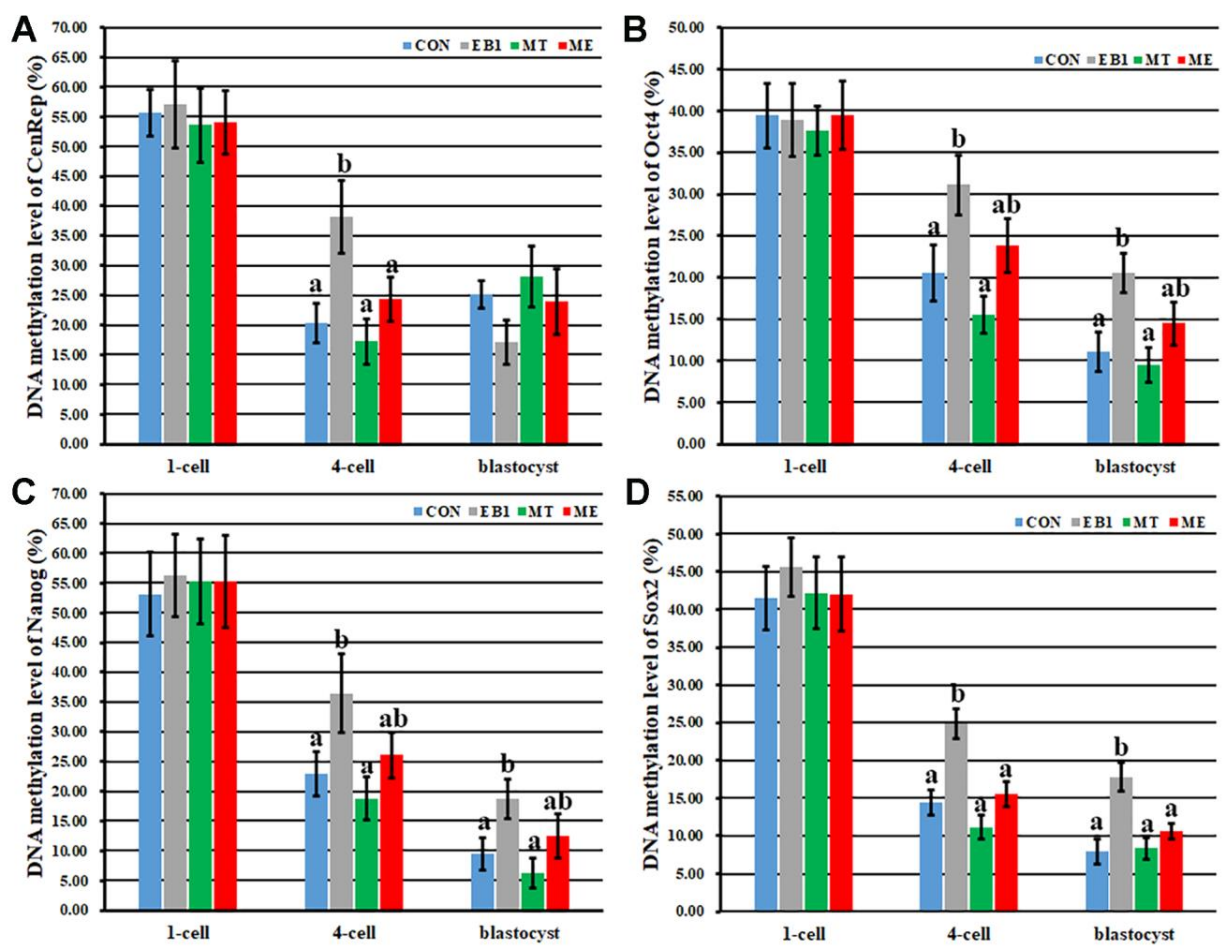
**Effect of melatonin on DNA methylation levels of genome and pluripotent genes in EB1-exposed embryos.** (**A**–**D**) DNA methylation levels of genome (*CenRep*), *Oct4*, *Nanog* and *Sox2*, respectively. CON, the control group. EB1, embryos treated with EB1. MT, embryos treated with melatonin. ME, embryos treated with both melatonin and EB1. ^a-b^Values for a given group in columns with different superscripts differ significantly (p < 0.05).

Then, DNA methylation reprogramming of genome (*CenRep*) and pluripotent genes (*Oct4*, *Nanog* and *Sox2*) was investigated. As shown in Figures 7, 8, the CON, EB1, MT and ME groups displayed the similar patterns of DNA demethylation and then remethylation of *CenRep* and DNA demethylation of *Oct4*, *Nanog* and *Sox2* during embryo development progress, however, *CenRep* DNA remethylation did not obviously occur at the blastocyst stage in the EB1 group. When compared with the CON group, the EB1 group displayed the significantly higher DNA methylation levels of *CenRep* at the 4-cell stage and *Oct4*, *Nanog* and *Sox2* at the 4-cell and blastocyst stages (P<0.05), while no significant DNA methylation differences of *CenRep*, *Oct4*, *Nanog* and *Sox2* were observed in the MT or ME group. Furthermore, DNA methylation levels of *CenRep* at the 4-cell stage and *Sox2* at the 4-cell and blastocyst stages were significantly lower in the ME group than the EB1 group (P<0.05). Thus, melatonin largely rescued the disrupted DNA methylation reprogramming in EB1-treated embryos.

**Figure 9:**
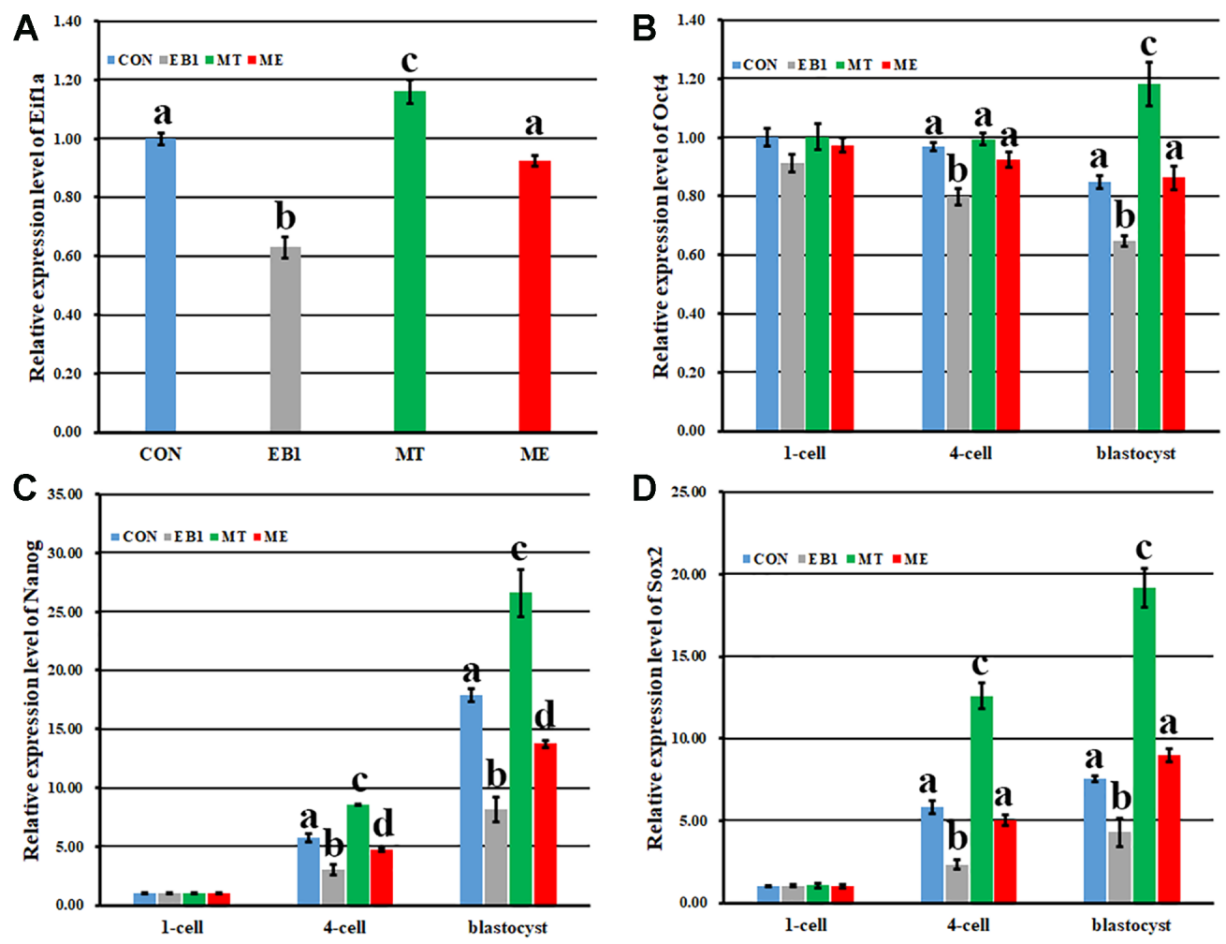
**Effect of melatonin on the expression of zygote genome activation and pluripotency related genes in EB1-exposed embryos.** (**A**–**D**) relative expression levels of *Eifla*, *Oct4*, *Nanog* and *Sox2*, respectively. CON, the control group. EB1, embryos treated with EB1. MT, embryos treated with melatonin. ME, embryos treated with both melatonin and EB1. ^a-d^Values for a given group in columns with different superscripts differ significantly (p < 0.05).

The expression patterns of genes related to early embryo development were further detected (Figure 9). As for zygotic activation related gene *Eif1a*, compared with the CON group, the EB1 group displayed the significantly decreased expression level, while the MT group showed the significantly increased transcription level (P<0.05), and the ME group took the similar expression level. Moreover, the expression level of *Eif1a* in the ME group was significantly higher than that in the EB1 group (P<0.05). When the expression patterns of *Oct4*, *Nanog* and *Sox2* were detected, similar expression trends were observed among the CON, EB1, MT and ME groups. However, compared with the CON group, the EB1 group showed the significantly lower expression levels of *Oct4*, *Nanog* and *Sox2* at the 4-cell and blastocyst stages, the MT group displayed the significantly higher transcription levels of *Oct4* at the blastocyst stage and *Nanog* and *Sox2* at the 4-cell and blastocyst stages, and the ME group only took the significantly lower expression levels of *Nanog* at the 4-cell and blastocyst stages (P<0.05). Moreover, the significantly upregulated expression levels of *Oct4*, *Nanog* and *Sox2* at the 4-cell and blastocyst stages were observed in the ME group compared with the EB1 group (P<0.05). These results displayed that melatonin ameliorated EB1-induced disturbed expression of genes in early embryos. Taken together, melatonin protected early embryos against the disrupted DNA methylation reprogramming and gene expression induced by EB1.

## DISCUSSION

**Figure 10:**
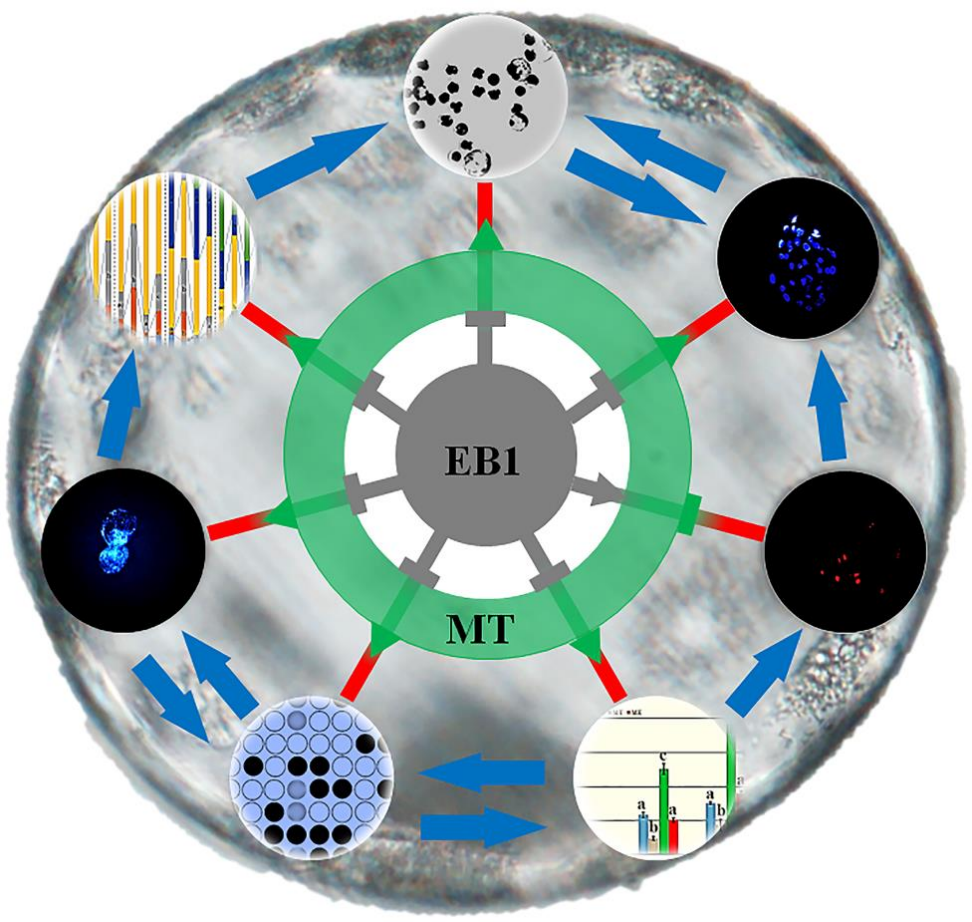
**Schematic representation of the protective role of melatonin against EB1 to ameliorate the development competence and quality of early embryos.** EB1 destroys nuclear remodeling, triggers apoptosis, disrupts DNA methylation reprogramming and gene expression, and further reduces the cleavage and blastocyst rates and blastocyst cell number of early embryos. Melatonin rescues EB1-mediated development defects and ameliorates the development competence and quality of early embryos.

It is known that exogenous factors influence embryo development, and recently, to evaluate the potential detrimental or beneficial effects of various environmental compounds on embryos has been gaining attention for human and animal reproductive health [3, 10, 24]. In this study, EB1 exerted the detrimental effects on the development competence and quality of porcine early embryos, and the potential mechanism could be that EB1 destroyed nuclear remodeling, triggered apoptosis, and disrupted DNA methylation reprogramming and gene expression. And more, melatonin was also proven to effectively protect against EB1-mediated defects during early embryo development (Figure 10).

Previous studies have shown that food or feed contaminated with toxins degrades early embryos [4, 25, 26]. As one emerging mycotoxin, EB1 could also have the potential detrimental effects on early embryos. Previous studies have shown that EB1 exerts cytotoxicity, arresting cell cycle or causing cell death [5, 8, 9]. In this study, EB1 was applied to treat porcine early embryos, and shown to reduce the cleavage and blastocyst rates of embryos, in consistent with exogenous hazardous compounds to impair embryo development [4, 25, 26]. Blastocyst cell number is also an objective index to reflect embryo cell proliferation and quality [27]. Here, the decreased blastocyst cell number displayed that EB1 destroyed embryo quality. In addition, EB1 exerted embryotoxicity in a dose and time dependent manner, suggesting that there is a cumulative toxic effect of EB1 to induce the detrimental impacts on embryos. Moreover, a recent study has shown that EB1 causes damage to mouse blastocysts [10]. Thus, EB1 is detrimental for the development competence and quality of early embryos.

During early embryogenesis, nuclear remodeling is the first step of early embryo development, determining the subsequent development competence of embryos [13, 28]. When embryos were treated with EB1, nuclear remodeling progress was inhibited, showing that sperm nucleus ineffectively carried out decondensation and formed pronucleus, and nuclear division and cytokinesis were also obviously delayed. This may be due to the interference of EB1 on the function of nuclear remodeling factors, as EB1 has been proven to destroy cell cycle [8]. As for the detail interaction between EB1 and nuclear remodeling factors, it needs to be investigated. Anyhow, these results reveal that EB1 delays or inhibits nuclear remodeling progress to further reduce the development competence of early embryos.

Apoptosis, which is mainly regulated by apoptosis or antioxidation related genes, is a key marker to evaluate embryo quality [15, 23, 29]. Thus, to examine embryo apoptosis status and the expression levels of apoptosis or antioxidation related genes can evaluate the detrimental effects of EB1 on early embryos. Notably, EB1 significantly triggered embryo apoptosis, further supporting that the increased apoptosis reduced blastocyst cell number in the EB1 group. The disrupted expression levels of apoptosis and antioxidant genes also confirmed this point, as EB1 could destroy the signaling pathway systems of antiapoptosis or antioxidation [7–9]. Moreover, EB1 has been displayed to trigger cell or mouse blastocyst apoptosis or death [8, 10, 30]. Accordingly, EB1 can act as one embryo apoptosis inducer and impairs embryo quality.

Gene expression levels are correlated with DNA methylation statuses of their promoters [31, 32]. Then, the degree of DNA methylation reprogramming, mainly regulated by *Dnmts* and *Tets*, determines the development competence of early embryos. During the development progress of early embryos, our study has demonstrated that *Dnmt1* transcripts gradually decrease from the 1-cell to morula stage with a slight increase at the blastocyst stage and take the role of DNA methylation maintenance during the development progress of early embryos, *Dnmt3a* expression levels are downregulated from the 1-cell to 8-cell stage, then gradually increase with the maximum at the blastocyst stage, and are responsible for DNA remethylation after the 4-cell stage, *Tet3* transcripts continuously decrease from the 1-cell to blastocyst stage with very low levels at the morula and blastocyst stages and mainly act as the role of DNA demethylation from the 1-cell to 4-cell stage, and *Tet1* expression levels steadily increase after the 4-cell stage with the highest levels at the blastocyst stage and work as the DNA demethylation role after the 4-cell stage [33]. In this study, similar expression patterns of *Dnmts* and *Tets* were also observed in early embryos. As for *Dnmt3a*, the transcripts also decreased from the 1-cell to 4-cell stage, and increased at the blastocyst stage compared with the 4-cell stage, and the cause could also be that *Dnmt3a*, as the maternal factor, degrades from the 1-cell stage to 4-cell stage, and then begins to express with the zygote genome activation at the 4-cell stage. Previous studies have also shown that DNA methylation is vulnerable to exogenous factors [12, 14, 34]. Here, the obviously increased DNA methylation levels of *CenRep* during zygote genome activation and *Oct4*, *Nanog* and *Sox2* at the 4-cell and blastocyst stages occurred in the EB1 group, as is evident from the upregulated expression of *Dnmt1* and *Dnmt3a* at the 4-cell stage and downregulated transcription of *Tet1* and *Tet3* at the 4-cell and blastocyst stages induced by EB1. Then, incomplete DNA methylation reprogramming reduced zygote genome activation and pluripotent gene expression, leading to the poor development and quality of embryos. As how EB1 disrupted the expression of DNA methylation reprogramming related genes, the possible pathway might be that EB1 changes enzyme activation, destroys energy metabolism, and then delays embryo development progress [8, 30]. Of course, the regulatory network of gene expression is complex, and further studies are needed. All in all, these results collectively demonstrate that EB1 exerts the obviously detrimental effects on the development competence and quality of early embryos.

Antioxidant or antiapoptotic system has been shown to take the critical protective role during early embryo development, and studies have also highlighted that melatonin can take the antioxidant and antiapoptotic effects to protect embryos against the damage induced by exogenous factors [22–24]. In this study, EB1 was proven to exert the detrimental effects on porcine early embryos, and the potential mechanism could be that EB1 destroyed nuclear remodeling, triggered embryo apoptosis and disrupted DNA methylation reprogramming. In our previous study, melatonin is shown to reduce apoptosis and enhance DNA methylation reprogramming in early embryos [21]. Then, it is wondered that whether melatonin can eliminate the detrimental effects of EB1 on early embryos through regulating nuclear remodeling, apoptosis and DNA methylation reprogramming. Here, this work demonstrated that when EB1-exposed embryos were treated with melatonin at the optimal concentration, which has been proven by investigating the early development of porcine cloned embryos [21], nuclear remodeling was obviously promoted, apoptosis was remarkedly reduced, DNA methylation reprogramming and gene expression were also largely ameliorated, and, importantly, the development competence and quality of embryos were similar to the control group, further supporting the potential of melatonin to protect embryos against damage caused by exogenous compounds. Previous studies have also displayed that melatonin can promote nuclear remodeling, reduce apoptosis, improve DNA methylation reprogramming and enhance gene expression [17, 20, 21]. Thus, melatonin could protect embryos against EB1-induced damage. As how melatonin antagonizes EB1 to rescue the defects during embryo development, especially the detail molecular mechanism such as the epigenetic modification mediated *Nrf2* or *NF-κB* pathway or DNA damage protection to regulate gene expression, it needs further studies [18, 19]. Overall, embryo damage induced by EB1 is effectively eliminated by melatonin.

In conclusion, this study displays that EB1 reduces the development competence and quality of porcine early embryos, and the underlying mechanism is that EB1 destroys nuclear remodeling, triggers apoptosis and further disrupts DNA methylation reprogramming and gene expression. Moreover, melatonin is shown to effectively protect against EB1-mediated defects during early embryo development.

## MATERIALS AND METHODS

Chemicals were purchased from Sigma Aldrich Corporation, and plasticware was obtained from Nunclon, unless otherwise stated.

All the experiments were approved and supervised by Animal Care Commission of Qingdao Agricultural University according to animal welfare laws, guidelines and policies.

### Oocyte *in vitro* maturation

Oocyte *in vitro* maturation has been reported in our previous study [35]. Briefly, ovaries were collected from a local slaughterhouse and transported to laboratory. Follicles were aspirated, and follicular contents were washed with HEPES-buffered Tyrode’s lactate. Cumulus-oocyte complexes (COCs) were recovered, washed and cultured in maturation medium. After 42 h, COCs were vortexed in hyaluronidase to remove cumulus cells. Only oocytes with the visible polar body, regular morphology and homogenous cytoplasm were used in the subsequent experiments.

### *In vitro* fertilization (IVF) and embryo culture, treatment and collection

The procedures for IVF and embryo culture have been described in our previous reports [13, 21]. Briefly, spermatozoa were diluted with modified Tris-buffered medium, and matured oocytes were transferred into fertilization medium and co-incubated with spermatozoa. Then, embryos were washed and cultured in porcine zygote medium-3 (PZM-3) for the subsequent development, and the cleavage and blastocyst rates were evaluated at 48 h and 156 h, respectively.

For embryo treatment, embryos were cultured in PZM-3 with 0, 5, 10, 25 or 50 μM EB1 for 12 h or 24 h, or co-incubated with 0.1 μM melatonin during the development progress [21].

For embryo collection, 1-cell, 4-cell and blastocyst embryos in the CON (untreated), EB1 (10 μM EB1 for 24 h), MT (0.1 μM melatonin) and ME (10 μM EB1 for 24 h and 0.1 μM melatonin) groups were collected at 12 h, 48 h and 156 h, respectively.

### Examination of nuclear remodeling and blastocyst cell number

Embryos at 2 h, 4 h, 6 h, 12 h, 18 h, 24 h and 156 h were treated with acidic Tyrode's solution to remove zona pellucida, fixed in 4% paraformaldehyde, and stained with Hoechst 33342, respectively [21]. Then, nuclear status and blastocyst cell number were examined, and the percentage of every nuclear status and average cell number per blastocyst were calculated.

### Assessment of embryo apoptosis

Detection of embryo apoptosis using the TUNEL method with an *In Situ* Cell Death Detection Kit (Roche) has been described in our previous work [29]. Briefly, blastocysts were fixed with 4% paraformaldehyde, permeabilized with 0.5% Triton X-100, incubated in the terminal deoxynucleotidyl transferase mediated dUTP nick end labeling reaction medium, and stained with Hoechst 33342. Then, the number of apoptotic cells per blastocyst was counted.

### Quantitative real-time PCR

Measurement of gene expression with quantitative real-time PCR has been applied in our previous study [33]. Briefly, total RNA was extracted from 50 pooled embryos at each stage using a RNeasy Micro Kit (Qiagen). Reverse transcription was performed using a PrimeScript^®^ RT Reagent Kit (TaKaRa). For quantitative real-time PCR, reactions were performed in the 96-well optical reaction plate using a SYBR^®^ Premix ExTaq^TM^ II kit (TaKaRa) and a 7500 Real-Time PCR System. For every sample, the cycle threshold (CT) values were obtained from three replicates. The primers were presented in Supplementary Table 1. Relative expression levels of genes were analyzed using the 2^−ΔΔCT^ method.

### Bisulfite sequencing

Bisulfite sequencing has been reported in our previous work [31]. Briefly, pooled embryos were digested and treated with sodium bisulfite to convert all unmethylated cytosine to uracil using an EZ DNA Methylation-Direct^TM^ Kit (Zymo Research). For samples of 500, 200 and 50 pooled embryos at the 1-cell, 4-cell and blastocyst stages, digestion was performed in the M-Digestion Buffer, cytosine to thymine conversation was carried out at 98° C for 10 min and 64° C for 2.5 h, and samples were desalted and purified. Subsequently, nested PCR was performed to amplify the target regions of genes using the primers described in Supplementary Table 2. The amplified products were verified, cloned into T-Vectors and sequenced. The sequenced results were analyzed to get the methylated (the black circle) and unmethylated (the white circle) CpG sites, and then DNA methylation level for every sequenced result was calculated by dividing the number of methylated CpG sites by the total number of CpG sites not including the mutated or single nucleotide polymorphism (SNP) CpG sites.

### Statistical analysis

Differences in data (mean ± SEM) were analyzed with the SPSS statistical software. Statistical analyses of data concerning embryo development, blastocyst cell number, nuclear remodeling, embryo apoptosis, gene expression and DNA methylation were performed with t-test when the comparison was made between two groups or one-way analysis of variance when there were more than two groups. For all analyses, differences were considered to be statistically significant when P<0.05, and ^a-d^different superscripts were applied to indicate statistically significant differences.

## Supplementary Material

Supplementary Tables

## References

[1] Peltoniemi O, Björkman S, Oropeza-Moe M, Oliviero C. Developments of reproductive management and biotechnology in the pig. Anim Reprod. 2019; 16:524–38. 10.21451/1984-3143-AR2019-0055 32435295 PMC7234181

[2] de Mouzon J, Chambers GM, Zegers-Hochschild F, Mansour R, Ishihara O, Banker M, Dyer S, Kupka M, Adamson GD. International committee for monitoring assisted reproductive technologies world report: assisted reproductive technology 2012†. Hum Reprod. 2020; 35:1900–13. 10.1093/humrep/deaa090 32699900

[3] Truong T, Gardner DK. Antioxidants improve IVF outcome and subsequent embryo development in the mouse. Hum Reprod. 2017; 32:2404–13. 10.1093/humrep/dex330 29136144

[4] Shin KT, Guo J, Niu YJ, Cui XS. The toxic effect of aflatoxin B1 on early porcine embryonic development. Theriogenology. 2018; 118:157–63. 10.1016/j.theriogenology.2018.06.002 29909260

[5] Fraeyman S, Croubels S, Devreese M, Antonissen G. Emerging fusarium and alternaria mycotoxins: occurrence, toxicity and toxicokinetics. Toxins (Basel). 2017; 9:228. 10.3390/toxins9070228 28718805 PMC5535175

[6] Rodríguez-Carrasco Y, Narváez A, Izzo L, Gaspari A, Graziani G, Ritieni A. Biomonitoring of enniatin B1 and its phase I metabolites in human urine: first large-scale study. Toxins (Basel). 2020; 12:415. 10.3390/toxins12060415 32580411 PMC7354432

[7] Novak B, Rainer V, Sulyok M, Haltrich D, Schatzmayr G, Mayer E. Twenty-eight fungal secondary metabolites detected in pig feed samples: their occurrence, relevance and cytotoxic effects *in vitro*. Toxins (Basel). 2019; 11:537. 10.3390/toxins11090537 31540008 PMC6784148

[8] Wätjen W, Debbab A, Hohlfeld A, Chovolou Y, Kampkötter A, Edrada RA, Ebel R, Hakiki A, Mosaddak M, Totzke F, Kubbutat MH, Proksch P. Enniatins A1, B and B1 from an endophytic strain of fusarium tricinctum induce apoptotic cell death in H4IIE hepatoma cells accompanied by inhibition of ERK phosphorylation. Mol Nutr Food Res. 2009; 53:431–40. 10.1002/mnfr.200700428 19065580

[9] Juan-García A, Ruiz MJ, Font G, Manyes L. Enniatin A1, enniatin B1 and beauvericin on HepG2: evaluation of toxic effects. Food Chem Toxicol. 2015; 84:188–96. 10.1016/j.fct.2015.08.030 26342765

[10] Huang CH, Wang FT, Chan WH. Enniatin B1 exerts embryotoxic effects on mouse blastocysts and induces oxidative stress and immunotoxicity during embryo development. Environ Toxicol. 2019; 34:48–59. 10.1002/tox.22656 30259633

[11] Bertero A, Moretti A, Spicer LJ, Caloni F. Fusarium molds and mycotoxins: potential species-specific effects. Toxins (Basel). 2018; 10:244. 10.3390/toxins10060244 29914090 PMC6024576

[12] Breton-Larrivée M, Elder E, McGraw S. DNA methylation, environmental exposures and early embryo development. Anim Reprod. 2019; 16:465–74. 10.21451/1984-3143-AR2019-0062 32435290 PMC7234019

[13] Li J, Huan Y, Xie B, Wang J, Zhao Y, Jiao M, Huang T, Kong Q, Liu Z. Identification and characterization of an oocyte factor required for sperm decondensation in pig. Reproduction. 2014; 148:367–75. 10.1530/REP-14-0264 25030891

[14] Meehan RR, Thomson JP, Lentini A, Nestor CE, Pennings S. DNA methylation as a genomic marker of exposure to chemical and environmental agents. Curr Opin Chem Biol. 2018; 45:48–56. 10.1016/j.cbpa.2018.02.006 29505975

[15] Yin SY, Chen L, Wu DY, Wang T, Huo LJ, Zhao S, Zhou J, Zhang X, Miao YL. Tris(1,3-dichloro-2-propyl) phosphate disturbs mouse embryonic development by inducing apoptosis and abnormal DNA methylation. Environ Mol Mutagen. 2019; 60:807–15. 10.1002/em.22322 31411769

[16] Loren P, Sánchez R, Arias ME, Felmer R, Risopatrón J, Cheuquemán C. Melatonin scavenger properties against oxidative and nitrosative stress: impact on gamete handling and *in vitro* embryo production in humans and other mammals. Int J Mol Sci. 2017; 18:1119. 10.3390/ijms18061119 28613231 PMC5485943

[17] Tian X, Wang F, Zhang L, Ji P, Wang J, Lv D, Li G, Chai M, Lian Z, Liu G. Melatonin promotes the *in vitro* development of microinjected pronuclear mouse embryos via its anti-oxidative and anti-apoptotic effects. Int J Mol Sci. 2017; 18:988. 10.3390/ijms18050988 28475125 PMC5454901

[18] Galano A, Tan DX, Reiter RJ. Melatonin: a versatile protector against oxidative DNA damage. Molecules. 2018; 23:530. 10.3390/molecules23030530 29495460 PMC6017920

[19] Korkmaz A, Rosales-Corral S, Reiter RJ. Gene regulation by melatonin linked to epigenetic phenomena. Gene. 2012; 503:1–11. 10.1016/j.gene.2012.04.040 22569208

[20] Yang M, Tao J, Wu H, Guan S, Liu L, Zhang L, Deng S, He C, Ji P, Liu J, Liu G. Aanat knockdown and melatonin supplementation in embryo development: involvement of mitochondrial function and DNA methylation. Antioxid Redox Signal. 2019; 30:2050–65. 10.1089/ars.2018.7555 30343588

[21] Qu J, Sun M, Wang X, Song X, He H, Huan Y. Melatonin enhances the development of porcine cloned embryos by improving DNA methylation reprogramming. Cell Reprogram. 2020; 22:156–66. 10.1089/cell.2019.0103 32207988

[22] Niu YJ, Zhou W, Nie ZW, Shin KT, Cui XS. Melatonin enhances mitochondrial biogenesis and protects against rotenone-induced mitochondrial deficiency in early porcine embryos. J Pineal Res. 2020; 68:e12627. 10.1111/jpi.12627 31773776

[23] Yao X, Jiang H, Gao Q, Li YH, Xu YN, Kim NH. Melatonin alleviates defects induced by zearalenone during porcine embryo development. Theriogenology. 2020; 151:66–73. 10.1016/j.theriogenology.2020.04.005 32311602

[24] Xu Y, Zhang KH, Sun MH, Lan M, Wan X, Zhang Y, Sun SC. Protective effects of melatonin against zearalenone toxicity on porcine embryos *in vitro*. Front Pharmacol. 2019; 10:327. 10.3389/fphar.2019.00327 31024301 PMC6460015

[25] Schoevers EJ, Santos RR, Fink-Gremmels J, Roelen BA. Toxicity of beauvericin on porcine oocyte maturation and preimplantation embryo development. Reprod Toxicol. 2016; 65:159–69. 10.1016/j.reprotox.2016.07.017 27474255

[26] Zhang Y, Jia RX, Pan MH, Lu Y, Cui XS, Kim NH, Sun SC. HT-2 toxin affects development of porcine parthenotes by altering DNA and histone methylation in oocytes matured *in vitro*. Theriogenology. 2017; 103:110–16. 10.1016/j.theriogenology.2017.07.052 28780481

[27] Guo S, Liu S, Bou G, Guo J, Jiang L, Chai Z, Cai M, Mu Y, Liu Z. Fetal bovine serum promotes the development of *in vitro* porcine blastocysts by activating the Rho-associated kinase signalling pathway. Reprod Fertil Dev. 2019; 31:366–76. 10.1071/RD18070 30253120

[28] Liu HJ, Liu RM. Dynamic changes in chromatin and microtubules at the first cell cycle in SCNT or IVF goat embryos. Cell Biol Int. 2018; 42:1401–09. 10.1002/cbin.11031 29993158

[29] Qu J, Wang X, Jiang Y, Lv X, Song X, He H, Huan Y. Optimizing 5-aza-2'-deoxycytidine treatment to enhance the development of porcine cloned embryos by inhibiting apoptosis and improving DNA methylation reprogramming. Res Vet Sci. 2020; 132:229–36. 10.1016/j.rvsc.2020.06.020 32619801

[30] Oliveira CA, Ivanova L, Solhaug A, Fæste CK. Enniatin B_1_-induced lysosomal membrane permeabilization in mouse embryonic fibroblasts. Mycotoxin Res. 2020; 36:23–30. 10.1007/s12550-019-00366-8 31264166

[31] Song X, Liu Z, He H, Wang J, Li H, Li J, Li F, Jiang Z, Huan Y. Dnmt1s in donor cells is a barrier to SCNT-mediated DNA methylation reprogramming in pigs. Oncotarget. 2017; 8:34980–91. 10.18632/oncotarget.16507 28380421 PMC5471028

[32] Calicchio R, Doridot L, Miralles F, Méhats C, Vaiman D. DNA methylation, an epigenetic mode of gene expression regulation in reproductive science. Curr Pharm Des. 2014; 20:1726–50. 10.2174/13816128113199990517 23888966

[33] Huan Y, Wang H, Wu Z, Zhang J, Liu Z, He H. The expression patterns of DNA methylation reprogramming related genes are associated with the developmental competence of cloned embryos after zygotic genome activation in pigs. Gene Expr Patterns. 2015; 18:1–7. 10.1016/j.gep.2015.04.001 25917378

[34] Ladd-Acosta C, Fallin MD. DNA methylation signatures as biomarkers of prior environmental exposures. Curr Epidemiol Rep. 2019; 6:1–13. 10.1007/s40471-019-0178-z 31032172 PMC6481677

[35] Huan Y, Xie B, Liu S, Kong Q, Liu Z. A novel role for DNA methyltransferase 1 in regulating oocyte cytoplasmic maturation in pigs. PLoS One. 2015; 10:e0127512. 10.1371/journal.pone.0127512 26009894 PMC4444208

