# Supplementary material for: Melatonin protects against defects induced by Enniatin B1 during porcine early embryo development
Source: Aging (Albany NY). 2021 Feb 11;13(4):5553–70. doi: 10.18632/aging.202484 (PMC7950273; doi:10.18632/aging.202484)
Supplement: Supplementary Tables [file aging-13-202484-s001.pdf]

SUPPLEMENTARY TABLES

Supplementary Table 1. Detail of primers for quantitative real-time PCR.

Gene	Primer sequence (5'-3')	Length (bp)	Accession number
Sod1	F: TTGGAGACCTGGGCAATGTGAC	184	NM_001190422
	R: CTTCCAGCATTTCCCGTCTTTGTA		
Gpx4	F: ATGCACGAATTCTCAGCCAAG	122	NM_214407
	R: GTGTAGTTTACCTCCGTCTTGCC		
Cat	F: AGGTGGGGCTCCAAATTACTAC	136	NM_214301
	R: CACCTGGGTGACATTATCTTCG		
Bcl2l1	F: CTGGTGGTTGACTTTCTCTCCTAC	119	NM_214285
	R: GTTTCCGCTTCTGATTCAGTCC		
Bax	F: CAGTAACATGGAGCTGCAGAGG	159	XM_003127290
	R: GCCTTGAGCACCAGTTTACTGG		
Caspase3	F: CAGTTGAGGCAGACTTCTTGTATG	149	NM_214131
	R: AGTAAGAATGTGCATAAGCTCAAGC		
Dnmt1	F: GCGTCTTGCAGGCTGGTCAGTA	152	NM_001032355
	R: CTTCTTATCATCGACCACGACGCT		
Dnmt3a	F: ATGTGGTTCGGAGACGGCAAGT	195	NM_001097437
	R: GCTCTCGTCGTTGTCATGGCA		
Tet1	F: TGTCGGCTTGGCAAGAAAGA	115	NM_001315772
	R: AGACCACTGTGCTGCCATTA		
Tet3	F: CCCCTACTCAGGAAATGAGGTCTC	126	XM_021087365
	R: CCCCAAATTCAGCCTCAAACTG		
Eif1a	F: AGATGAGGCTAGAAGTCTGAAGGC	113	NM_001243218
	R: CAATGTCATCAAACTGGATTTCATC		
Oct4	F: GAAGGTGTTCAGCCAAACGAC	185	NM_001113060
	R: CGATACTTGTCCGCTTTC		
Nanog	F: CCTCCATGGATCTGCTTATTC	209	NM_001129971
	R: CATCTGCTGGAGGCTGAGGT		
Sox2	F: AACCAGAAGAACAGCCCAGAC	155	NM_001123197
	R: TCCGACAAAAGTTTCCACTCG		
18s	F: AATCTCGGGTGGCTGAACGC	143	NR_002170
	R: CCGTTCTTAGTTGGTGGAGCGAT		

Supplementary Table 2. Detail of primers for bisulfite sequencing.

Gene	Primer sequence (5'-3')	Length (bp)	Accession number
CenRep	F: GGTATTGTTGTTTGTTTGGTGATT	231	Z75640
	R: AAAATTTATTCCTCAAACCCAATTT		
Oct4	Outer	409	NC_010449
	F: ATTAGATTTGTGTGAGGATTTGAGAG		
	R: AAAACCCAATAAAACCAAAACTCTC		
	Inner	249	
	F: GTGTATAGAGTAGTGGAGAGGG		
	R: CCCAATCCCACCCACTAA		
Nanog	Outer	518	EF_522119
	F: TGAATTGGAGATTTAAAGGAG		
	R: TAAAATCATAAAAATCTCCTCC		
	Inner	498	
	F: GGAGATTTAAAGGAGTTTTAGGTTAAGAAA		
	R: TCTCCTCCAAATATTAAAAATATCAAAAA		
Sox2	Outer	461	NC_010455
	F: GTGTTTGTAAAAGGGGGAAAGTAG		
	R: CCGCAACAATAAAATTACCACC		
	Inner	321	
	F: AAGGGAGAGAAGTTTGAGTTTTAGG		
	R: AACTCCGTCTCCATCATATTATACATAC		

Supplementary Table 3. Development of early embryos treated with different concentration EB1 for 24 h.

EB1 (μM)	No. embryos (Rep)	No. embryos cleaved (% ± SEM)	No. blastocysts (% ± SEM)	Blastocyst cell number (mean ± SEM)*
0	192 (5)	155 (80.78 ± 1.78)[a]	40 (20.96 ± 0.73)[a]	41 ± 2 (n=40)[a]
5	187 (5)	140 (74.81 ± 2.80)[ab]	34 (17.96 ± 1.12)[a]	36 ± 2 (n=33)[ab]
10	198 (5)	140 (70.48 ± 1.09)[bc]	22 (10.73 ± 1.03)[b]	31 ± 2 (n=21)[b]
25	201 (5)	136 (67.97 ± 1.94)[c]	12 (6.11 ± 1.24)[c]	31 ± 3 (n=10)[b]
50	188 (5)	122 (65.04 ± 3.05)[c]	10 (5.61 ± 0.98)[c]	28 ± 3 (n=7)[b]

[a-c]Values in the same column with different superscripts differ significantly (P<0.05).
*Blastocyst cell number, less than 16, was not included.

Supplementary Table 4. Development of early embryos treated with 10 μM EB1 for different time.

Time (h)	No. embryos (Rep)	No. embryos cleaved (% ± SEM)	No. blastocysts (% ± SEM)	Blastocyst cell number (mean ± SEM)*
0	142 (3)	115 (81.23 ± 2.25)[a]	29 (20.20 ± 2.24)[a]	41 ± 3 (n=29)[a]
12	144 (3)	107 (74.72 ± 2.78)[a]	23 (15.86 ± 1.55)[a]	35 ± 3 (n=23)[ab]
24	159 (3)	110 (69.43 ± 1.92)[b]	15 (9.59 ± 0.90)[b]	30 ± 3 (n=14)[b]

[a-b]Values in the same column with different superscripts differ significantly (P<0.05).
*Blastocyst cell number, less than 16, was not included.

Supplementary Table 5. Effect of melatonin on the development of EB1-exposed embryos.

Group	No. embryos (Rep)	No. embryos cleaved (% ± SEM)	No. blastocysts (% ± SEM)	Blastocyst cell number (mean ± SEM)*
CON	200 (5)	159 (79.80 ± 1.10)[a]	43 (21.75 ± 1.39)[a]	42 ± 2 (n=42)[a]
EB1	180 (5)	125 (69.57 ± 1.60)[b]	19 (10.67 ± 1.61)[b]	30 ± 3 (n=17)[b]
MT	174 (5)	147 (84.41 ± 1.23)[c]	50 (28.68 ± 1.71)[c]	48 ± 2 (n=49)[c]
ME	206 (5)	154 (75.25 ± 1.41)[d]	38 (18.26 ± 0.90)[a]	38 ± 1 (n=37)[a]

CON referred to embryos untreated. EB1 referred to embryos treated with Enniatin B1. MT referred to embryos treated with melatonin. ME referred to embryos treated with both melatonin and enniatin B1.

[a-d]Values in the same column with different superscripts differ significantly (P<0.05).

*Blastocyst cell number, less than 16, was not included.

Supplementary Table 6. Effect of melatonin on nuclear remodeling of EB1-exposed embryos during the first cell cycle.

Time (h)	Group	No. embryos (Rep)	No. embryos unfertilized (%±SEM)	No. embryos with sperm nuclear condensation (%±SEM)	No. embryos with sperm nuclear decondensation (%±SEM)	No. embryos with paternal pronucleus (%±SEM)	No. embryos with nuclear division (%±SEM)	No. embryos with cytokinesis (%±SEM)
2	CON	44 (3)	7 (15.28±1.39)	15 (34.72±1.39)[a]	11 (25.00±1.60)	11 (25.00±1.60)[a]	0 (0.00±0.00)	0 (0.00±0.00)
	EB1	43 (3)	6 (13.60±1.10)	22 (51.21±2.63)[b]	10 (23.36±2.33)	5 (11.84±0.66)[b]	0 (0.00±0.00)	0 (0.00±0.00)
	MT	59 (3)	9 (15.29±0.50)	10 (16.88±1.09)[c]	17 (28.65±2.34)	23 (39.18±2.92)[c]	0 (0.00±0.00)	0 (0.00±0.00)
	ME	45 (3)	7 (15.05±1.49)	15 (33.97±2.39)[a]	11 (24.80±2.72)	12 (26.18±1.96)[a]	0 (0.00±0.00)	0 (0.00±0.00)
4	CON	65 (3)	10 (15.41±1.27)	15 (23.26±1.53)[a]	13 (20.06±1.35)	27 (41.27±3.35)[a]	0 (0.00±0.00)	0 (0.00±0.00)
	EB1	63 (3)	9 (14.31±0.39)	25 (39.67±0.83)[b]	15 (23.77±2.53)	14 (22.26±1.73)[b]	0 (0.00±0.00)	0 (0.00±0.00)
	MT	60 (3)	9 (15.08±0.79)	4 (6.61±1.47)[c]	12 (20.11±1.06)	35 (58.20±1.91)[c]	0 (00.00±0.00)	0 (0.00±0.00)
	ME	58 (3)	8 (13.68±0.98)	14 (24.11±0.45)[a]	13 (22.45±1.23)	23 (39.76±0.90)[a]	0 (0.00±0.00)	0 (0.00±0.00)
6	CON	70 (3)	8 (11.62±1.71)	11 (15.51±0.89)[a]	17 (24.32±0.35)[a]	34 (48.55±2.14)[a]	0 (0.00±0.00)	0 (0.00±0.00)
	EB1	73 (3)	8 (10.87±0.67)	24 (32.91±1.13)[b]	22 (30.04±0.73)[b]	19 (26.19±1.63)[b]	0 (0.00±0.00)	0 (0.00±0.00)
	MT	66 (3)	8 (12.10±1.24)	5 (7.84±1.93)[c]	7 (10.59±3.04)[c]	46 (69.48±2.24)[c]	0 (0.00±0.00)	0 (0.00±0.00)
	ME	54 (3)	6 (10.84±0.60)	9 (17.26±1.37)[a]	17 (31.19±0.61)[b]	22 (40.70±0.70)[d]	0 (0.00±0.00)	0 (0.00±0.00)
12	CON	55 (3)	8 (14.47±0.71)	4 (7.15±1.25)[ac]	7 (12.88±1.82)[ac]	36 (65.50±1.17)[ac]	0 (0.00±0.00)[a]	0 (0.00±0.00)
	EB1	86 (3)	10 (11.86±1.86)	21 (24.64±2.55)[b]	27 (30.97±2.81)[b]	28 (32.53±1.49)[b]	0 (0.00±0.00)[a]	0 (0.00±0.00)
	MT	73 (3)	9 (12.29±2.26)	2 (2.62±1.32)[a]	6 (8.40±2.78)[a]	52 (71.44±3.11)[a]	4 (5.25±2.64)[b]	0 (0.00±0.00)
	ME	76 (3)	10 (13.03±2.19)	9 (12.05±1.07)[c]	14 (18.16±2.27)[c]	43 (56.77±3.94)[c]	0 (0.00±0.00)[a]	0 (0.00±0.00)
18	CON	65 (3)	9 (13.73±2.29)	1 (1.59±1.59)[a]	1 (1.59±1.59)[a]	25 (38.58±2.34)	24 (36.85±1.79)[a]	5 (7.66±1.47)[a]
	EB1	77 (3)	8 (10.77±2.10)	11 (14.10±1.37)[b]	20 (25.98±1.01)[b]	29 (37.68±1.16)	9 (11.47±1.25)[b]	0 (0.00±0.00)[b]
	MT	58 (3)	7 (12.01±0.25)	0 (0.00±0.00)[a]	1 (1.39±1.39)[a]	21 (36.60±2.36)	20 (34.64±3.46)[a]	9 (15.36±1.82)[c]
	ME	70 (3)	8 (11.44±1.45)	2 (2.85±1.43)[a]	6 (8.30±3.85)[a]	27 (38.80±3.48)	23 (32.87±0.96)[a]	4 (5.75±1.48)[a]
24	CON	79 (3)	9 (11.29±1.32)	0 (0.00±0.00)	1 (1.45±1.45)[a]	18 (23.01±1.54)[ac]	15 (18.66±1.27)[a]	36 (45.59±1.26)[a]
	EB1	68 (3)	7 (10.21±0.71)	0 (0.00±0.00)	10 (15.06±2.54)[b]	25 (36.46±3.30)[b]	7 (10.21±0.71)[b]	19 (28.07±0.97)[b]
	MT	93 (3)	10 (10.72±0.22)	0 (0.00±0.00)	1 (0.93±0.93)[a]	16 (17.03±1.68)[a]	19 (20.70±3.46)[a]	47 (50.62±1.56)[c]
	ME	82 (3)	8 (9.80±1.33)	0 (0.00±0.00)	4 (4.79±0.94)[a]	23 (27.98±1.18)[c]	15 (18.30±1.97)[a]	32 (39.14±1.25)[d]

CON referred to embryos untreated. EB1 referred to embryos treated with Enniatin B1. MT referred to embryos treated with melatonin. ME referred to embryos treated with both melatonin and enniatin B1.

[a-d]Values at the same time in the same column with different superscripts differ significantly (P<0.05).
